# Breast self-examination programmes in the trial of early detection of breast cancer: ten year findings.

**DOI:** 10.1038/bjc.1993.315

**Published:** 1993-07

**Authors:** R. Ellman, S. M. Moss, D. Coleman, J. Chamberlain

**Affiliations:** DH Cancer Screening Evaluation Unit, Institute of Cancer Research, Sutton, Surrey, UK.

## Abstract

Programmes of education in breast self-examination with specialist clinics for self-referral were introduced in two health districts around 1980. Combining the results from the two centres showed no reduction in mortality from breast cancer over the following 10 years but the mortality was low in one of the centres whilst in the other it was higher than in four geographically separate comparison centres in which there was similar careful monitoring of breast cancer incidence and mortality. Because this was not a randomised controlled trial and lacked a uniform treatment protocol, biases may be responsible for the differences observed, but it is also possible that BSE education with annual reinforcement contributed to the breast cancer mortality reduction seen in one district. The overall conclusion however is that the value of breast self-examination remains unproven.


Br. J. Cancer (1993), 68, 208-212 © Macmillan Press Ltd., 1993

### Breast self-examination programmes in the trial of early detection of breast cancer: ten year findings

R. Ellman, S.M. Moss, D. Coleman & J. Chamberlain

*UK Trial of Early Detection of Breast Cancer Group*. Trial Co-ordinating Cente, DH Cancer Screening Evaluation Unit, Institute of Cancer Research, Section of Epidemiology, D Block, Cotswold Road, Sutton, Surrey SM2 5NG, UK.*

**Summary** Programmes of education in breast self-examination with specialist clinics for self-referral were introduced in two health districts around 1980. Combining the results from the two centres showed no reduction in mortality from breast cancer over the following 10 years but the mortality was low in one of the centres whilst in the other it was higher than in four geographically separate comparison centres in which there was similar careful monitoring of breast cancer incidence and mortality. Because this was not a randomised controlled trial and lacked a uniform treatment protocol, biases may be responsible for the differences observed, but it is also possible that BSE education with annual reinforcement contributed to the breast cancer mortality reduction seen in one district. The overall conclusion however is that the value of breast self-examination remains unproven.

Interest in the UK Trial of Early Detection of Breast Cancer (TEDBC) has tended to focus on the effects of screening by mammography but the trial also includes two centres, Nottingham and Huddersfield, in which women were encouraged to practise breast self-examination (BSE). As an attempt to assess prospectively the effects of BSE encouragement on breast cancer mortality within a defined population it is unique.

It was found that, while the two screening centres presented a very similar pattern of breast cancer mortality, the two centres offering teaching in BSE were dissimilar. Details of the method of the trial (UK Trial of Early Detection of Breast Cancer Group, 1981) and of the effects of screening and BSE education on breast cancer mortality (UK Trial of Early Detection of Breast Cancer Group, in press) have already been published. The purpose of this paper is to explore the possible explanations for the contrast between the BSE centres.

### Method

### *Recruitment*

At the start of the trial the first cohort of women aged 45 to 64 years was recruited from the lists of all general practitioners serving the different populations. Their date of entry to the trial was the date on which they were first invited by letter to a BSE class, between 1979 and 1981. The date of entry in comparison centres was arbitrarily fixed as January 1st 1980. In Nottingham classes and clinics were held at a fixed central hospital location whereas in Huddersfield alternative local community venues were offered. Non-attenders in Nottingham were initially sent a further appointment for a BSE class but only 8% responded and second invitations were therefore discontinued. In Huddersfield a low attendance rate in the first year led to increased efforts to influence non-attenders. They were sent two more invitations at 18 month intervals and these included leaflets on BSE and details of the self-referral clinics which women could attend whether or not they had been to the BSE class. Approximately half the non-attenders as well as the attenders were sent calendars annually on which they were asked to record their monthly BSE examination. Training courses were organised to encourage community nursing staff to teach BSE wherever the opportunity might arise. Both centres publicised the programme through local newspapers and by holding open days.

### *Classes*

At the BSE class a short film was shown, demonstrating a systematic BSE method, and a talk was given by a specially trained nurse, health educator or surgeon, but there were no individual demonstrations or base-line examinations of the breasts.

### *Clinics*

The open-access clinics provided breast examination by a specially trained nurse and mammography was performed provided the patient had not had a mammogram within the last year. Women might instead choose to consult their GPs in which case no record was sent to the TEDBC unless the episode led to biopsy.

### *Breast pathology and treatment*

During the first 7 years information on pathological features was collected for all breast biopsies and personal and treatment details were extracted from clinical case-notes.

After the fieldwork period information on further breast cancers was mainly obtained from cancer registrations in the NHS central registries where records of all the women in the Trial have been flagged. As cancer registration notification is incomplete and delayed, analysis of incident cancers is limited to cases occurring within the first 7 years of trial entry.

There were differences between the centres in surgical and pathology practice, a major difference being in the proportion of breast cancer cases where nodal status was assessed, and the extent of such assessment. Consequently operable tumours are classified according to the maximum diameter reported by the pathologist rather than by nodal status. There was some variation between laboratories on whether measurements were made on fresh or fixed, or both fixed and sectioned material.

### *Deaths*

Notifications of deaths and cancer registrations are received from the NHS Central Registry. The breast cancer deaths

Correspondence: R. Ellman, DH Cancer Screening Evaluation Unit, Institute of Cancer Research, Section of Epidemiology, D Block, Cotswold Road, Sutton, Surrey SM2 5NG, UK.

*Other members and co-authors from the following hospitals: Cookridge Hospital, Leeds (C. Joslin), Pennine Screening Office, Bradford (G. Harris, J. Philip), Nottingham City Hospital (R. Blamey, J. Caseldine, C. Elston, E. Roebuck, A. Mitchell), Ninewells Hospital & Medical School, Dundee (J. Swanson Beck), Southmead Hospital, Westbury-on-Trym (P. Bradfield), Department of Public Health Medicine, Stoke-on-Trent (M. Summerley).

Received 13 July 1992; accepted 25 January 1993.

included in this paper are those in which breast cancer was recorded as the underlying cause of death on the death certificate and had been first diagnosed after entry to the trial. Follow up was censored at 31st December 1989 or within 10 years of entry to the trial, whichever was earlier. Deaths from breast cancers diagnosed between 7 and 10 years from entry are included in mortality analysis though not in the incidence data.

### *Analysis*

Mortality rates were calculated, using woman-years of follow up as denominator since staggered entry led to variable length of follow-up (UK Trial of Early Detection of Breast Cancer Group, in press). Expected rates were calculated adjusting first for age within 5 year bands and year since trial entry, and second by multiplying this figure by the pre-trial breast cancer standardised mortality ratio (SMR) taking the pooled rate over the previous 10 years for all six centres as standard. The risk of death in each BSE centre has been calculated relative to that of the combined comparison centres. Two-tailed probability-values greater than 0.05 are considered non-significant.

### Results

### *Attendance at classes and clinics*

Attendance at classes was markedly lower in Huddersfield than in Nottingham and declined with age (Table I). Use of the special breast clinics was higher in the first year after trial entry than in subsequent years and was higher in Huddersfield than in Nottingham, especially in later years. It declined with age.

### *Benign biopsies and breast cancers*

The benign biopsy rate was raised 2.1-fold in Huddersfield but only 1.2-fold in Nottingham compared with that of the combined comparison centres. The younger women had the higher rates, but were less affected by the trial intervention.

In contrast to benign biopsy the incidence of malignancy tended to rise with age at entry and was slightly lower in Huddersfield than in Nottingham, presenting a 7% increase relative to the combined comparison centres *vs* a 13% increase in Nottingham. Both BSE centres reported increased detection of tumours less than 21 mm, the rate in Huddersfield being slightly lower than that in Nottingham.

Neither BSE centre experienced much fall in the incidence of cancers over 20 mm or with fixation or distant metastasis (Figure 1). Huddersfield, with a slightly raised rate in the early years which later 'crossed over' that of the comparison centres, follows the expected pattern for a successful early detection programme (Day *et al*., 1989). There was no such cross-over pattern in Nottingham but the cumulative rate was lower throughout.

### *Treatment*

Breast conserving operations and the use of hormone therapy became increasingly popular over the period of the trial. Chemotherapy, as an adjuvant for early stage disease, was however little used other than in Huddersfield. Table II shows that Huddersfield was not only outstanding in the use made of chemotherapy (in the form of short course monochemotherapy or perioperative poly-chemotherapy) but also made greater use of breast conserving operations and hormone therapy (tamoxifen). Nottingham used relatively little of any of these forms of treatment or of radiotherapy.

### *Mortality*

Mortality in the combined BSE centres is the same as in the combined comparison centres but there are marked differences between the two BSE centres (Table III). The relative risk in Huddersfield, adjusted for age and period, is 0.80 and is only slightly affected by further adjustment for pre-trial differences in breast cancer mortality between the centres. The relative risk in Nottingham is 1.23 but is reduced to 1.14 by pre-trial SMR adjustment as the breast cancer mortality was relatively high in 1969–78. The difference between Huddersfield and Nottingham, without pre-trial adjustment, is statistically significant ($P < .05$), while those between the comparison centres are not.

Mortality was consistently lower in attenders than in non-attenders but in Huddersfield both groups had low rates, whereas in Nottingham they both had high rates (Table IV),

### Discussion

The first question to consider is whether the mortality difference between Huddersfield and Nottingham could be

**Table I** Class attendance, clinic utilisation, benign biopsy and cancer detection in relation to age at entry (percentages of women)

| | *Women total* | *Class attendance in Yr 1* | *Clinic attendance in Yr 1* | *Clinic attendance in Yrs 2–7* | *Benign biopsy in Yrs 1–7* | *Breast cancer detection in Yrs 1–7* |
|---|---|---|---|---|---|---|
| *45–49 years* | | | | | | |
| Huddersfield | 5040 | 34.5% | 2.6% | 7.4% | 2.30% | 1.31% |
| Nottingham | 11144 | 56.3% | 2.3% | 3.5% | 1.18% | 1.16% |
| Comparison centres | 31553 | – | – | – | 1.20% | 1.08% |
| *50–54 years* | | | | | | |
| Huddersfield | 5866 | 33.4% | 2.5% | 5.1% | 1.50% | 1.16% |
| Nottingham | 10476 | 54.4% | 1.7% | 2.4% | 0.59% | 1.35% |
| Comparison centres | 32138 | – | – | – | 0.58% | 1.14% |
| *55–59 years* | | | | | | |
| Huddersfield | 6423 | 31.7% | 1.8% | 4.5% | 0.75% | 1.42% |
| Nottingham | 10933 | 52.8% | 1.7% | 1.7% | 0.58% | 1.49% |
| Comparison centres | 35644 | – | – | – | 0.34% | 1.31% |
| *60–64 years* | | | | | | |
| Huddersfield | 5253 | 26.1% | 1.3% | 2.8% | 0.72% | 1.22% |
| Nottingham | 8436 | 48.0% | 1.1% | 1.0% | 0.40% | 1.45% |
| Comparison centres | 26742 | – | – | – | 0.30% | 1.27% |
| *Total* | | | | | | |
| Huddersfield | 22582 | 31.4% | 2.1% | 4.9% | 1.28% | 1.28% |
| Nottingham | 40989 | 53.1% | 1.7% | 2.2% | 0.71% | 1.35% |
| Comparison centres | 126077 | – | – | – | 0.61% | 1.20% |

**Figure 1** Cumulative incidence rates for tumours of size >20 mm or with fixation or distant metastasis.

due to differences in their BSE programmes. The programmes were basically similar and it was Nottingham which achieved the higher invitation response rate. However, women could be influenced by the programme without attending and attendance may be a poor guide to whether women perform BSE satisfactorily and whether, having discovered a worrying sign, they refer themselves promptly for investigation.

We know from an interview survey (Calnan et al., 1983) that in Nottingham 28% of women were practising BSE satisfactorily shortly before they were invited to participate, and that a year later BSE practice among those who attended the class had increased to 47% whereas in non-attenders it remained at 33%. Over the same period practice increased from 24% to 28% in a comparison centre. Unfortunately similar information is not available for Huddersfield or for later years. The greater use made of self-referral clinics in Huddersfield in the later trial years may be an indication that Huddersfield achieved a more sustained level of BSE awareness through the issue of calendars than could be achieved through media publicity.

The tendency towards a smaller size of invasive lesion at detection was more evident in Nottingham which argues against the hypothesis that BSE was responsible for the lower mortality in Huddersfield but the different measuring methods may invalidate comparisons. The direction of bias is uncertain; though fixation results in shrinkage, examination under the microscope may reveal that infiltration by tumour is more extensive than is apparent to the naked eye.

The fact that Nottingham had a greater increase in breast cancer incidence than Huddersfield and the small size of these increases relative to the 41–51% increase observed in the screening centres might be considered to rule out the possibility that the mortality reduction in Huddersfield is due to the early detection programme. However it can be argued that success may be critically dependent on the stage at which the faster growing cancers are picked up and that breast awareness and prompt self-referral by women who develop early signs of cancer may be important. A long lead time afforded to slow growing cancers, may raise detection rates considerably, but be less critical.

Artefacts of the study design must next be considered. In both centres publicity about the BSE programme could reach women before they were personally invited to attend, so that some cancers diagnosed early as a result of the programme may have been excluded as 'pre-trial' cases. If such women approached the Trial unit they were permitted to attend but were excluded from analysis. In Nottingham three such women who attended before invitation and who died of breast cancer have been excluded, but no such deaths occurred in Huddersfield. Those who consulted their GP as a result of publicity are not individually distinguishable.

The inclusion of all pre-trial cases diagnosed within 12 months of date of entry brings the crude breast cancer mortality risk in Huddersfield closer to that of the comparison centres (RR from 0.81 to 0.89) but does not bring it to unity while the crude relative risk for Nottingham (RR 1.2) remains unaffected.

**Table IV** Age/period adjusted breast cancer mortality rates over first 10 years in those who attended BSE education in the first year and those who did not

| | *Deaths in attenders* | | *Deaths in non-attenders* | |
|---|---|---|---|---|
| | *No.* | *Per 1000 w.y.* | *No.* | *Per 1000 w.y.* |
| Huddersfield | 20 | 3.03 | 71 | 4.49 |
| Nottingham | 108 | 5.74 | 124 | 7.39 |
| Comparison centres | | | 646 | 5.18 |

**Table II** Women undergoing various treatments for management of newly diagnosed breast cancers during first 7 years (percentages of breast cancer patients)

| *Centre* | *Total breast cancers* | *Breast conserving operation*[a] | *Chemotherapy* | *Endocrine therapy* | *Radiotherapy* |
|---|---|---|---|---|---|
| Huddersfield | 289 | 30.4% | 39.4% | 53.6% | 50.5% |
| Nottingham | 555 | 13.3% | 2.3% | 18.0% | 16.6% |
| Comparison centres | 1528 | 19.4% | 6.0% | 26.4% | 41.4% |

[a]Includes quadrantectomy, lumpectomy, wide excision, tylectomy, and subcutaneous mastectomy.

**Table III** Breast cancer deaths over 10 years of follow-up and expected deaths (based on the pooled rates of the six centres)

| *Centre* | *Observed no.* | *Expected Age and period adjusted* | *Expected With pre-trial SMR adjustment* |
|---|---|---|---|
| Huddersfield | 91 | 116.4 | 115.3 |
| Nottingham | 232 | 193.2 | 206.8 |
| Comparison centres | 646 | 659.3 | 641.1 |

Risks relative to the combined comparison centres:

| | *Without pre-trial adjustment (95% CI)* | *With pre-trial SMR adjustment (95% CI)* |
|---|---|---|
| Huddersfield | 0.80 (0.64–0.99) | 0.78 (0.61–0.96) |
| Nottingham | 1.23 (1.06–1.43) | 1.14 (0.95–1.35) |
| Combined BSE centres | 1.07 (0.93–1.22) | 1.01 (0.86–1.17) |

Differences in underlying breast cancer incidence could also lead to differences in mortality. The adjustment whereby expected deaths for each district were multiplied by the pre-trial breast cancer SMRs was intended to counter bias due to differing underlying incidence rates but may fail to do so where boundary and demographic changes have occurred. It may also be relevant that the SMRs are based on all breast cancer deaths irrespective of the length of survival whereas the observed deaths in a study with limited follow up are biased towards exclusion of deaths after late recurrence; a centre where women traditionally referred themselves early would have a lower observed mortality in a study such as ours even though its SMR was 100%.

Trends in breast cancer mortality are shown in Figure 2. These are for cancers which may have been diagnosed at any time in the past, whereas trial death rates refer to women who were apparently disease-free at entry. The trends confirm that some improvement has occurred in Huddersfield. The improvement was not seen in women over 74 but was as marked in women under 45 as in those who were in the trial age range. This suggests that if the BSE programme is responsible, the benefit may be from its general effect of increasing breast awareness rather than an effect restricted to those directly targeted.

During the TEDBC period the results of clinical trials of mastectomy *vs* breast conservation were beginning to influence management (Gazet *et al.*, 1985), and some of the centres were themselves running further trials. It is unlikely that differences in extent of surgery shown in Table II would alter mortality although clearly having a major effect on morbidity and possibly an effect on early self-referral behaviour. However, both adjuvant chemotherapy and tamoxifen are now known to improve survival (Early Breast Cancer Trialists' Collaborative Group, 1992). On the

**Figure 2** Breast cancer mortality. Age standardised to E&W population.

assumption that such therapy reduces mortality of cancers without local fixation or distant metastasis by about 25% we can estimate the extent to which equal use of these agents in all centres might have reduced differences between centres (Table V). The difference between Nottingham and Huddersfield is only slightly reduced. We have also looked for bias affecting ascertainment of breast cancer deaths by reassessing the cause of death in all breast cancer patients in the trial. None was found (UK Trial of Early Detection of Breast Cancer Group, 1991).

These findings reported here are seemingly at variance with two studies carried out in Nottingham (based on the population of North Nottingham in addition to that included in this trial) which appeared to show a benefit from the BSE programme (Locker *et al.*, 1989). They compared prognostic factors of cases detected after the BSE campaign began with those in a pre-trial series of cases notified by the local cancer registry and also used a case-control design to compare the BSE class attendance of women who died of breast cancer with that of age-matched controls. Ascertainment of cases was not, however, as thorough in the pre-trial period as during the trial and lead time and length biases also make interpretation difficult. Likewise the case-control method is prone to selection bias which has been found to exaggerate the estimate of benefit in studies of mammographic screening trials (Moss *et al.*, 1992; Gulberg *et al.*, 1991). The case-control method may also have been biased by the exclusion from consideration of cancers detected in the first 3 months after invitation.

We conclude that the favourable mortality in Huddersfield may be partly due to the programme but it is also influenced by biases which cannot be corrected for. The difficulties experienced in trying to evaluate the BSE programmes naturally raise the question of the validity of inferences about the screening centres since they were compared with the same, geographically separate populations. The consistency of mortality reduction between the two screening centres (UK Trial of Early Detection of Breast Cancer Group, in press) and the similarity with results from elsewhere together with the conformity of the cancer incidence patterns to theoretical expectations in regard to prevalence/incidence ratios, size distribution at detection and incidence rates in the intervals between screens strongly suggest that women in the screening centres did benefit. The size of benefit cannot however be accurately estimated in view of the sources of bias which have been discussed.

Unfortunately the only current BSE trials which might provide more conclusive evidence, are trials in Moscow, Leningrad and East Berlin (Koroltchouk, 1990) in which factories are randomised, and these trials are jeopardised by political upheavals. The suggestion that a BSE programme with annual, personal, postal reminders may be effective, especially in societies where late cancer presentation is common, could be further pursued. A trial based solely on postal BSE encouragement in Belfast (Turner *et al.*, 1984) showed some impact on behaviour although it was too small and

**Table V** Effect of assuming that 25% of deaths occurring in non-advanced cases not given adjuvant therapy could have been avoided

| | Total deaths (n) | Total deaths (per 1000 w.y.) | Deaths among non-advanced cases not given adjuvant[a] (a) | Expected deaths assuming adjuvant prevents 25% (n − ¼ a) | (per 1000 w.y.) |
|---|---|---|---|---|---|
| Huddersfield | 91 | 0.424 | 21 | 85.75 | 0.400 |
| Nottingham | 232 | 0.629 | 96 | 208 | 0.564 |
| Comparison centres | 646 | 0.531 | 200 | 596 | 0.490 |

| | Crude RR (95% CI) | Expected RR with use of adjuvant for all (95% CI) |
|---|---|---|
| Comparison centres | 1.00 | 1.00 |
| Huddersfield | 0.80 (0.04–0.99) | 0.82 (0.65–1.02) |
| Nottingham | 1.18 (1.02–1.38) | 1.15 (0.98–1.35) |

[a]Cases without local fixation or distant metastases, not given tamoxifen or chemotherapy.

short-lived to show an effect on breast cancer mortality. Perhaps a larger and more sustained randomised controlled trial of BSE encouragement, based solely on postal reminders, should be attempted in a situation where the cost of mammographic screening is prohibitive.

This work was funded by the Department of Health but does not necessarily represent the views of the Department.

### References

CALNAN, M.W., CHAMBERLAIN, J. & MOSS, S. (1983). Compliance with a class teaching breast self-examination. *J. Epidemiol. Community Health*, **37**, 264-270.

DAY, N.E., WILLIAMS, D.R.R. & KAW, K.T. (1989). Breast cancer screening programmes: the development of a monitoring and evaluation system. *Br. J. Cancer*, **59**, 954-958.

EARLY BREAST CANCER TRIALISTS' COLLABORATIVE GROUP (1992). Systemic treatment of early breast cancer by hormonal, cytotoxic or immune therapy. *Lancet*, **339**, 1-15, 72-84.

GAZET, J.-C., RAINSBURY, R.M., FORD, H.T., POWLES, T.J. & COOMBES, R.C. (1985). Survey of treatment of primary breast cancer in Great Britain. *BMJ*, **290**, 1793-1795.

GULBERG, B., ANDERSSON, I., JANZON, L. & RANSTAM, J. (1991). Screening mammography. *Lancet*, **337**, 244.

KOROLTCHOUK, V. (1990). The USSR/Germany/WHO BSE Study and Global Strategies for the Control of Breast Cancer. In *Cancer Screening*, Miller, A.B., Chamberlain, J., Day, N.E., Hakama, M. & Prorok, P.C. (eds) pp. 56-67. UICC Project on Evaluation of Screening for Cancer. Cambridge University Press: Cambridge.

LOCKER, A.P., CASELDINE, J., MITCHELL, A.K., BLAMEY, R.W., ROEBUCK, E.J. & ELSTON, C.W. (1989). Results from a seven-year programme of breast self-examination in 89,010 women. *Br. J. Cancer*, **60**, 401-405.

MOSS, S.M., SUMMERLEY, M.E., THOMAS, B.A., ELLMAN, R. & CHAMBERLAIN, J. (1992). A case-control evaluation of the effect of breast cancer screening in the UK Trial of Early Detection of Breast Cancer. *J. Epid. Commun. Med.*, **46**, 362-364.

TURNER, J., ROY, D., IRWIN, G., BLANEY, R., ODLING-SMEE, W. & MACKENZIE, G. (1984). Does a booklet on breast self-examination improve subsequent detection rates? *Lancet*, **ii**, 337-339.

UK TRIAL OF EARLY DETECTION OF BREAST CANCER GROUP (1981). Trial of Early Detection of Breast Cancer: description of method. *Br. J. Cancer*, **44**, 618-627.

UK TRIAL OF EARLY DETECTION OF BREAST CANCER GROUP (1991). Verification of the cause of death in the UK Trial of Early Detection of Breast Cancer. *Br. J. Cancer*, **64**, 1151-1156.

UK TRIAL OF EARLY DETECTION OF BREAST CANCER GROUP. Breast cancer mortality after ten years in the UK Trial of Early Detection of Breast Cancer. *The Breast* (in press).


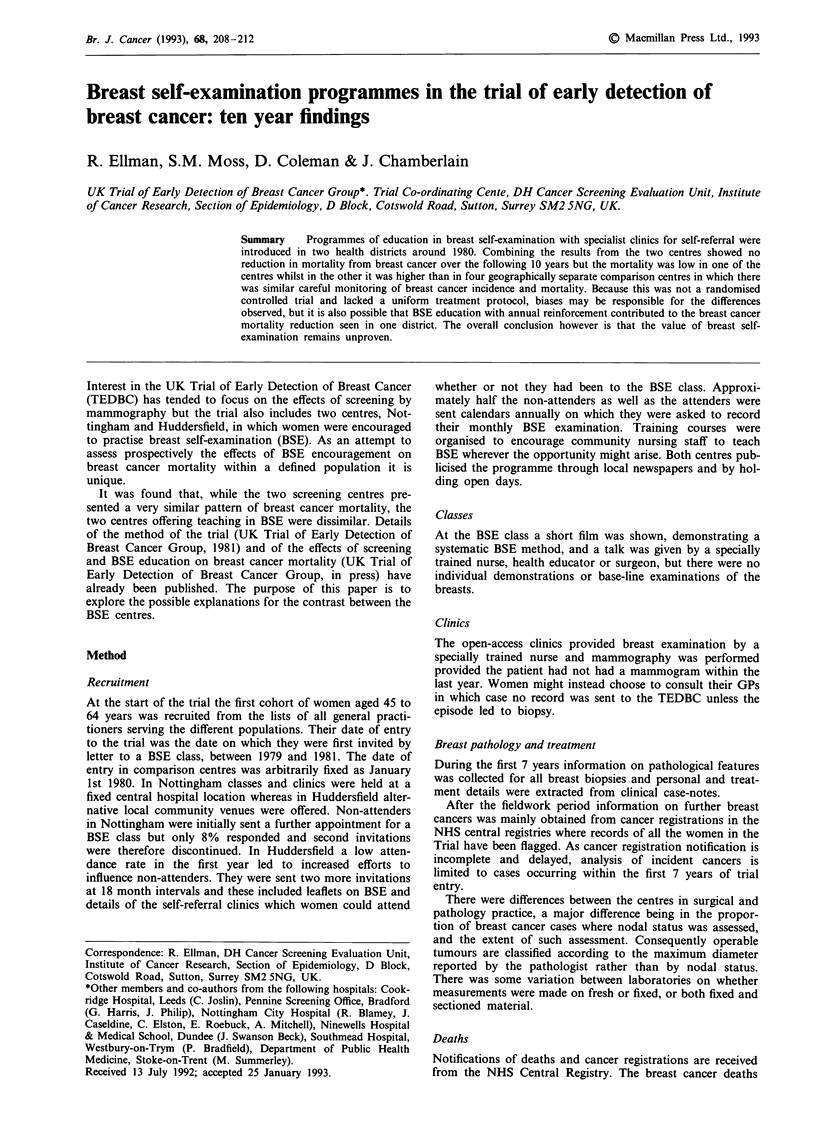

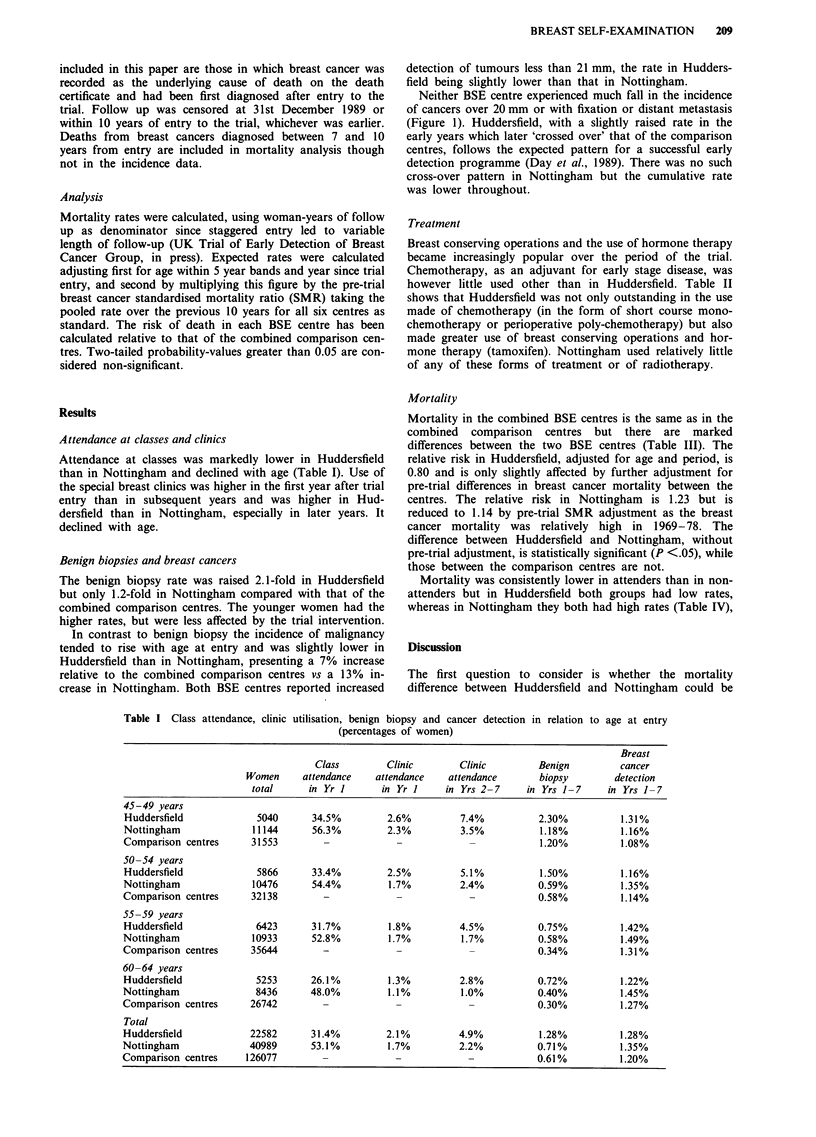

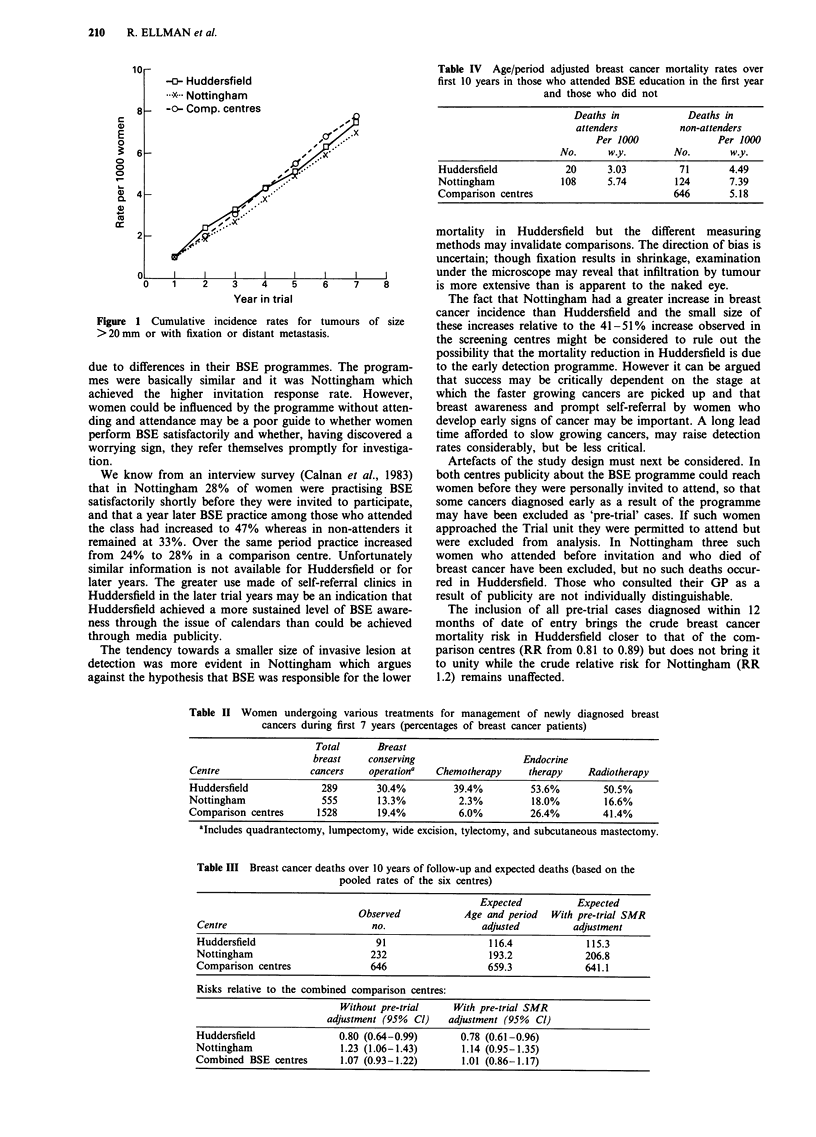

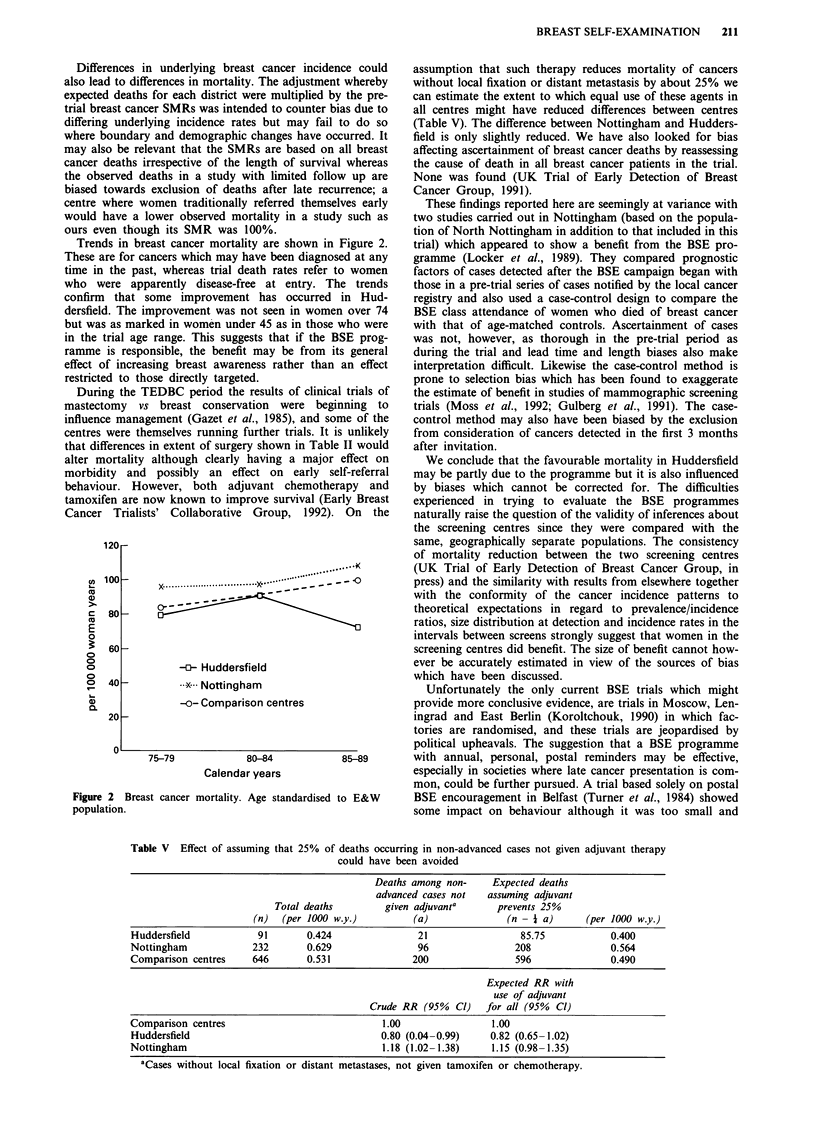

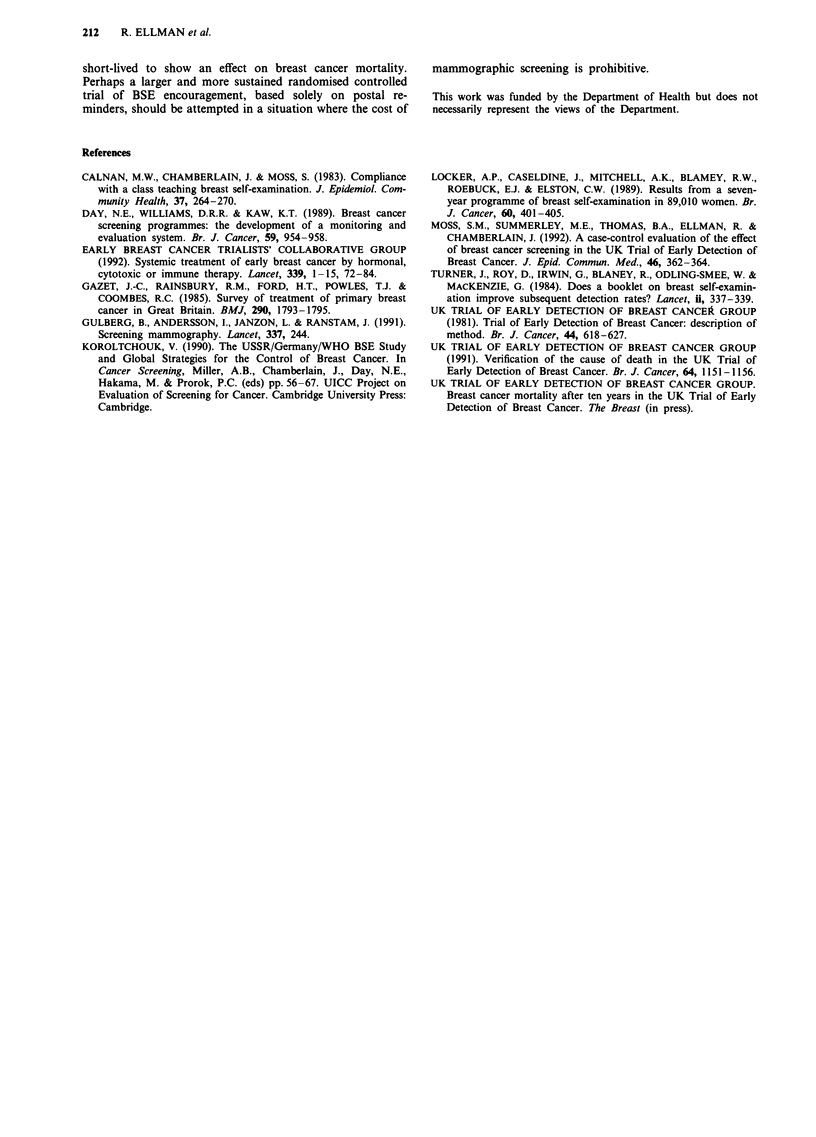


## References

[OCR_00626] Calnan M. W., Chamberlain J., Moss S. (1983). Compliance with a class teaching breast self examination.. J Epidemiol Community Health.

[OCR_00631] Day N. E., Williams D. R., Khaw K. T. (1989). Breast cancer screening programmes: the development of a monitoring and evaluation system.. Br J Cancer.

[OCR_00641] Gazet J. C., Rainsbury R. M., Ford H. T., Powles T. J., Coombes R. C. (1985). Survey of treatment of primary breast cancer in Great Britain.. Br Med J (Clin Res Ed).

[OCR_00646] Gullberg B., Andersson I., Janzon L., Ranstam J. (1991). Screening mammography.. Lancet.

[OCR_00658] Locker A. P., Caseldine J., Mitchell A. K., Blamey R. W., Roebuck E. J., Elston C. W. (1989). Results from a seven-year programme of breast self-examination in 89,010 women.. Br J Cancer.

[OCR_00664] Moss S. M., Summerley M. E., Thomas B. T., Ellman R., Chamberlain J. O. (1992). A case-control evaluation of the effect of breast cancer screening in the United Kingdom trial of early detection of breast cancer.. J Epidemiol Community Health.

[OCR_00670] Turner J., Blaney R., Roy D., Odling-Smee W., Irwin G., Mackenzie G. (1984). Does a booklet on breast self-examination improve subsequent detection rates?. Lancet.

